# Serum interleukin-6 (Il-6) monitoring in idiopathic multicentric Castleman disease: important value in patients receiving non-anti-Il-6 therapy

**DOI:** 10.1186/s40364-026-00943-x

**Published:** 2026-06-01

**Authors:** Zi-han Yang, Yu-han Gao, Si-yuan Li, Miao-yan Zhang, An-an Li, Hao-yi Xu, Lu Zhang, Jian Li

**Affiliations:** 1 Department of Hematology, Peking Union Medical College Hospital, Chinese Academy of Medical Sciences & Peking Union Medical College, Beijing, China; 2 State Key Laboratory of Complex, Severe, and Rare Diseases, Institute of Clinical Medicine, Peking Union Medical College Hospital, Chinese Academy of Medical Sciences & Peking Union Medical College, Beijing, China

**Keywords:** Idiopathic multicentric Castleman disease, Serum interleukin-6, Non-anti-IL-6 therapy, Treatment response, Biomarker

## Abstract

Idiopathic multicentric Castleman disease (iMCD) is a rare hematologic disorder characterized by a systemic cytokine storm. Interleukin-6 (IL-6) is central to iMCD pathogenesis and IL-6-targeted therapy is the preferred first-line treatment, yet most patients still receive non-anti-IL-6 regimens. We conducted a single-center retrospective study of patients with iMCD to evaluate the clinical utility of serum IL-6 monitoring—at baseline and longitudinally—with particular emphasis on those receiving non-anti-IL-6 therapy. Among 149 patients (660 IL-6 measurements; 76 male/73 female; median age 45 years), 34 received anti-IL-6 therapy and 115 received non-anti-IL-6 therapy. Baseline IL-6 differed across clinical and pathologic subtypes and correlated negatively with hemoglobin and albumin, and positively with platelet count, C-reactive protein (CRP), and immunoglobulin G (IgG). Baseline IL-6 did not predict biochemical response. IL-6 dynamics were uninformative in the anti-IL-6 group; however, in the non-anti-IL-6 group, IL-6 declined significantly after treatment, with greater decreases in responders than in non-responders. Among initial responders, a directional trend toward rising IL-6 levels was observed prior to biochemical PD identification, whereas persistently suppressed IL-6 was observed in sustained remission. Early IL-6 change predicted subsequent biochemical response; at 1 month, a ΔlogIL-6 threshold of − 0.16 achieved 91.7% sensitivity and 92.3% specificity. These findings support serum IL-6 monitoring as a useful tool for response assessment and early prediction in iMCD, especially among patients receiving non-anti-IL-6 therapies.

**Supplementary Information:**

The online version contains supplementary material available at 10.1186/s40364-026-00943-x.

## To the Editor:

Idiopathic multicentric Castleman disease (iMCD) is a rare, heterogeneous, and life-threatening hematologic disorder characterized by systemic inflammation, with interleukin-6 (IL-6) recognized as a key driver [1]. Three clinical subtypes have been described: iMCD-thrombocytopenia, anasarca, fever, reticulin fibrosis / renal dysfunction, organomegaly (TAFRO); idiopathic plasmacytic lymphadenopathy (IPL); and not otherwise specified (NOS) [2]. IL-6 inhibition is the recommended first-line therapy. Notably, both siltuximab and tocilizumab cause serum IL-6 elevation, either through the formation of long-lived IL-6-siltuximab complexes or by blocking IL-6R-mediated IL-6 clearance, and serum IL-6 is not recommended for assessing treatment response [3–5]. In fact, the accessibility of IL-6 inhibition in real-world practice remains limited, and there is no recommendation for monitoring serum IL-6 in patients receiving non-anti-IL-6 therapies [6–8]. In this context, we conducted a single-center retrospective study to assess baseline associations and longitudinal trajectories of IL-6, with a focus on non-IL-6-inhibited patients to evaluate IL-6 as a marker and predictor of treatment response.

We consecutively enrolled patients diagnosed with iMCD according to the Castleman Disease Collaborative Network (CDCN) consensus criteria, and who received treatment and had serum IL-6 monitoring at Peking Union Medical College Hospital (PUMCH) from July 2016 through June 2025 [1]. Patients treated with regimens containing siltuximab or tocilizumab were classified into the ‘anti-IL-6 therapies’ group, whereas patients who did not receive any IL-6-targeted drug were assigned to the ‘non-anti-IL-6 therapies’ group. Severe iMCD and treatment response was assessed according to the CDCN criteria [4]. Patients achieving biochemical PR or CR after the current treatment were classified as responders, whereas those with biochemical SD or PD were classified as non-responders.

A total of 149 iMCD patients were included, contributing 685 IL-6 measurements (Table S1). Thirty-four patients received anti-IL-6 therapies and 115 received non-anti-IL-6 therapies. The two groups were generally comparable in demographic and clinical characteristics, except for hemoglobin and albumin (Table S2). Importantly, baseline serum IL-6 levels were comparable between the two groups.

**Fig. 1:**
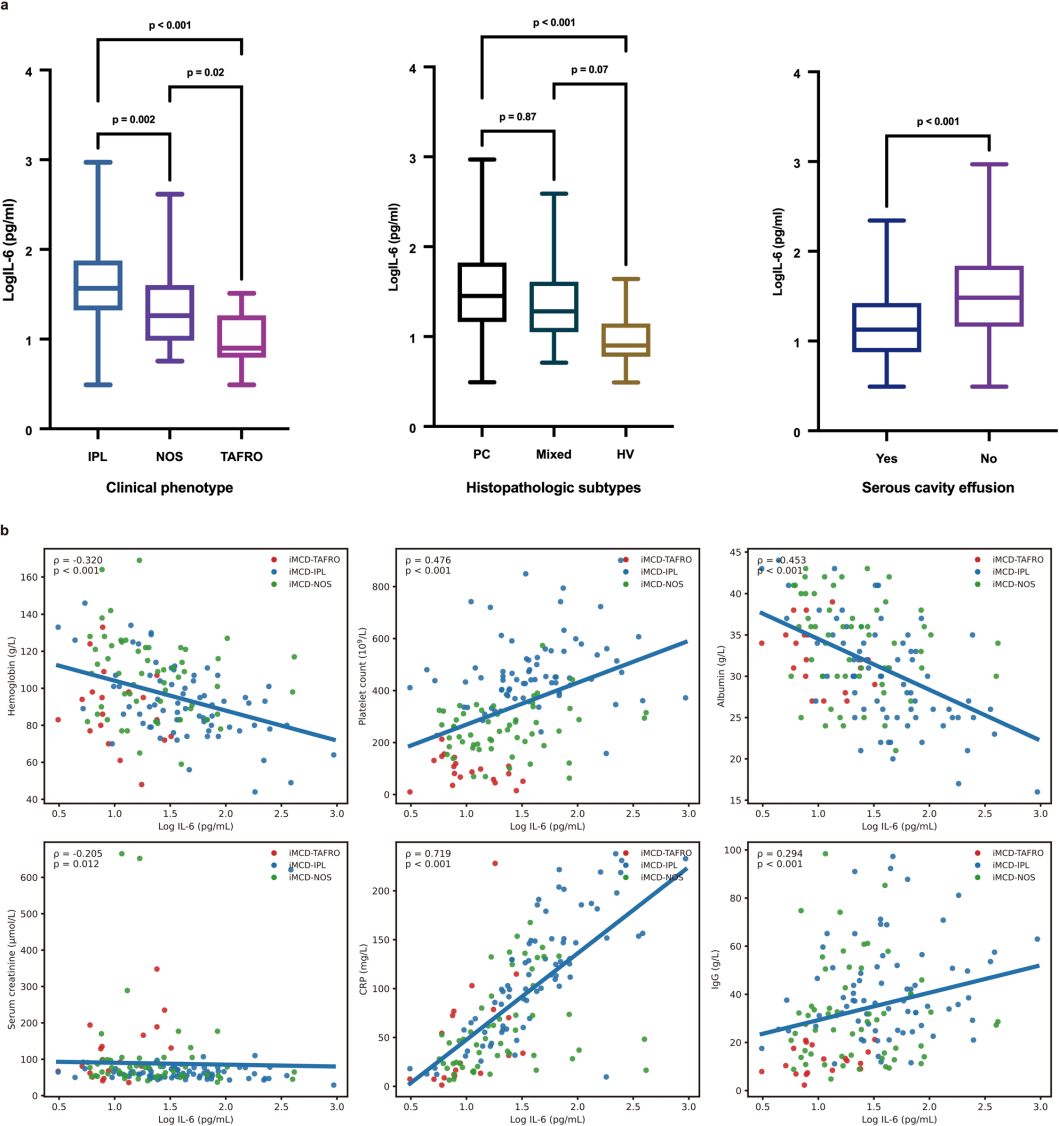
Associations between baseline serum IL-6 and clinical / laboratory characteristics in iMCD (**a**) Serum IL-6 levels differed significantly across subtypes: by clinical phenotype, median (interquartile range) logIL-6 levels were highest in iMCD-IPL 1.6 (1.3-1.9), intermediate in iMCD-NOS 1.3 (1.0-1.6), and lowest in iMCD-TAFRO 0.9 (0.8-1.3); by histopathology, logIL-6 was higher in the plasmacytic 1.5 (1.2-1.8) than in the hyaline vascular subtype 0.9 (0.8-1.1), with the mixed subtype intermediate 1.3 (1.1-1.6) and not significantly different from either. Patients without serous cavity effusion had higher logIL-6 1.5 (1.2-1.8) than those with effusion 1.1 (0.9-1.4). (**b**) Baseline log10 IL-6 correlates positively with CRP, platelet count, and IgG, and negatively with hemoglobin, albumin, and serum creatinine. iMCD, idiopathic multicentric Castleman disease; TAFRO, thrombocytopenia, anasarca, fever, reticulin fibrosis / renal dysfunction, organomegaly; IPL, idiopathic plasmacytic lymphadenopathy; NOS, not otherwise specified; PC, plasmacytic; HV, hyaline vascular; IL-6, interleukin-6; CRP, C-reactive protein; IgG, immunoglobulin G; logIL-6, log10-transformed IL-6

To evaluate the role of baseline IL-6 in profiling iMCD, we analyzed its associations with clinical characteristics and laboratory tests (Fig. 1 and Table S3). Baseline IL-6 differed by both subtype and histopathology—iMCD-IPL > iMCD-NOS > iMCD-TAFRO, and plasmacytic > mixed > hyaline vascular. Patients without serous cavity effusion had significantly higher IL-6 than those with effusion. Baseline IL-6 correlated positively with CRP, platelets, and IgG, and inversely with albumin and hemoglobin. No significant differences in baseline IL-6 levels were observed between responders and non-responders in either treatment group (Figure S1).

**Fig. 2:**
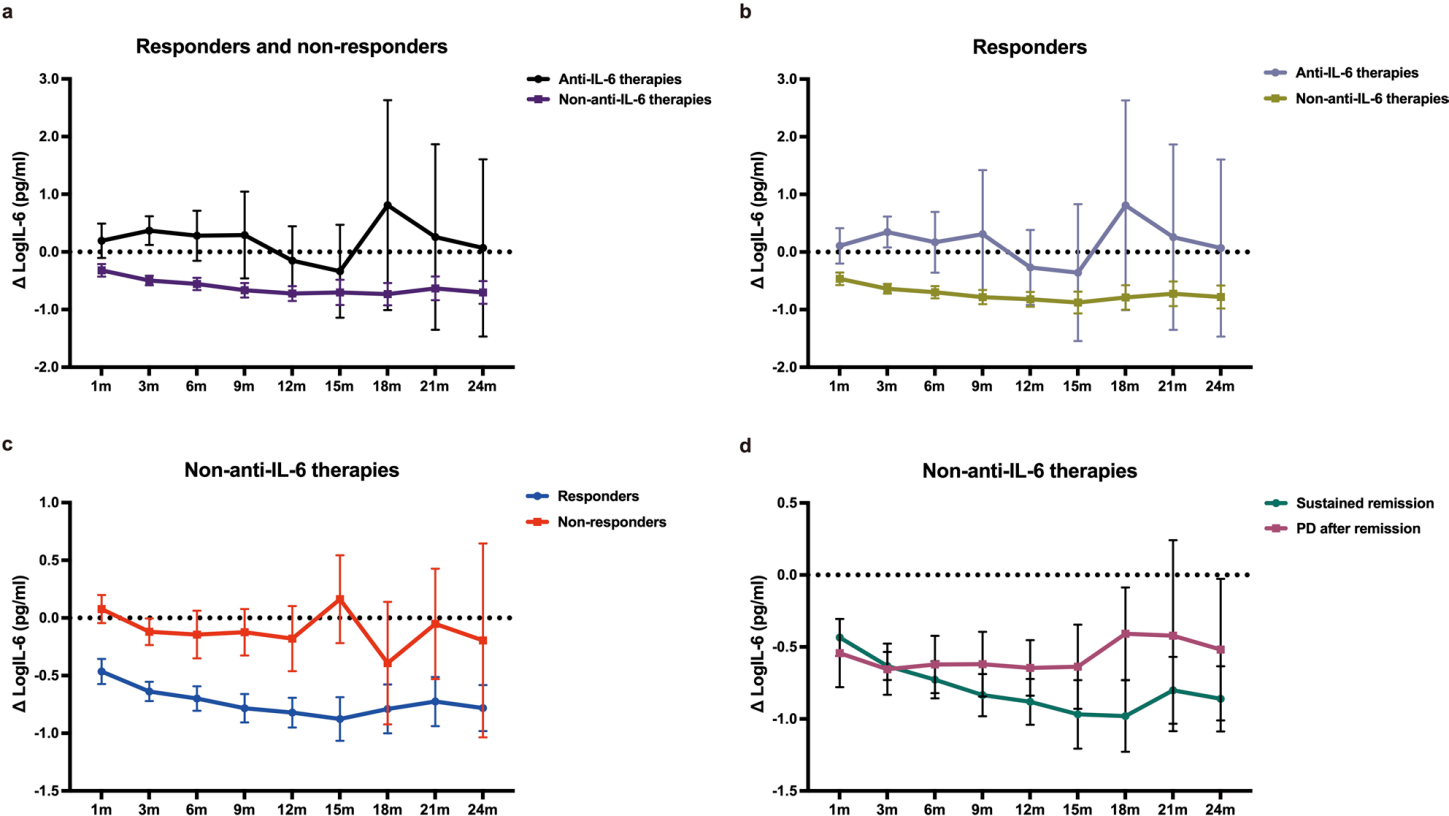
Trajectories of serum IL-6 levels under anti-IL-6 and non-anti-IL-6 therapies (**a**) Longitudinal change in IL-6 (ΔlogIL-6) for all treated iMCD patients. Anti-IL-6 therapy resulted in a modest increase in IL-6 levels above baseline (positive ΔlogIL-6) at most follow-up points (Table S3-1). In contrast, non-anti-IL-6 therapy led to a significant decrease from baseline (negative ΔlogIL-6) across the follow-up points. (**b**) Among responders only, anti-IL-6 therapies again caused an overall increase in IL-6 relative to baseline, whereas non-anti-IL-6 therapies exhibited a significant and sustained decrease. (**c**) In patients receiving non-anti-IL-6 therapies, IL-6 levels decreased more substantially in responders than in non-responders, with statistically significant differences emerging as early as 1 month and persisting through 15 months (Table S3-3). (**d**) Among patients who initially responded to non-anti-IL-6 therapies, those who maintained sustained remission continued to show suppressed IL-6 levels, whereas those who later experienced PD exhibited an early post-treatment IL-6 decline followed by a rebound. PD, progressive disease; IL-6, interleukin-6; ΔlogIL-6, the change in log10-transformed IL-6 relative to baseline

We next compared the IL-6 trajectories in the two treatment groups (Fig. 2 and Table S4). In the anti-IL-6 therapy group, IL-6 increased above baseline at most follow-up points, without significant relationship with biochemical response. In contrast, in the non-anti-IL-6 therapy group, IL-6 changes were significantly associated with response, with responders showing a significantly greater decrease in IL-6 compared to non-responders. Especially, after excluding iMCD-TAFRO patients, these associations were preserved (Figure S2 and Table S5). Moreover, in patients receiving non-anti-IL-6 therapies who initially responded, those who later experienced PD showed a rising trend in IL-6 levels up to 3 months prior to biochemical PD identification (Figure S3). However, within the limits of sample size and visit density, serum IL-6 showed a directionally consistent but statistically non-significant trend toward predicting exacerbation in a matched multivariable conditional logistic regression model. (Table S6). In non-anti-IL-6-treated iMCD-IPL patients, ΔlogIL-6 significantly mirrored changes in key labs (Figure S4). Notably, in two patients receiving non-anti-IL-6 treatment, transient CRP elevations occurred without corresponding deterioration in IL-6 or other clinical markers (Figure S5). Moreover, in patients on non-anti-IL-6 therapies, 1-month ΔlogIL-6 predicted later biochemical response, with a − 0.16 cutoff giving 92% sensitivity and 92% specificity (Figure S6).

Baseline serum IL-6 appeared more informative for disease characterization than for response prediction [9–11]. In contrast, longitudinal IL-6 monitoring provided information in patients receiving non-anti-IL-6 therapies. Early IL-6 decline may serve as a marker of treatment efficacy, potentially preceding or complementing conventional biochemical assessment—potentially earlier than CRP normalization [12].

In conclusion, monitoring IL-6 trajectories is of limited value during anti-IL-6 therapy, but is informative in patients receiving non-anti-IL-6 regimens. This study supports incorporating IL-6 monitoring into baseline disease characterization of iMCD, response assessment and early prediction for non-anti-IL-6-treated iMCD patients.

## Supplementary Information

Below is the link to the electronic supplementary material.

Supplementary Material 1

## Data Availability

The datasets used and/or analysed during the current study are available from the corresponding author on reasonable request.

## Abbreviations

iMCD: Idiopathic multicentric Castleman disease
IL-6: Interleukin-6
TAFRO: Thrombocytopenia, anasarca, fever, reticulin fibrosis / renal dysfunction, organomegaly
IPL: Idiopathic plasmacytic lymphadenopathy
NOS: Not otherwise specified
CDCN: Castleman Disease Collaborative Network
PUMCH: Peking Union Medical College Hospital
