# Supplementary material for: Serum interleukin-6 (Il-6) monitoring in idiopathic multicentric Castleman disease: important value in patients receiving non-anti-Il-6 therapy
Source: Biomark Res. 2026 Jun 1;14:55. doi: 10.1186/s40364-026-00943-x (PMC13227809; doi:10.1186/s40364-026-00943-x)
Supplement: Supplementary file 1 — Supplementary Material 1 [file 40364_2026_943_MOESM1_ESM.docx]

**Supplementary Methods**

**Study population**

This single-center retrospective cohort study consecutively enrolled patients with iMCD, diagnosed according to the Castleman Disease Collaborative Network (CDCN) consensus criteria, who received treatment and had serum IL-6 monitoring at Peking Union Medical College Hospital (PUMCH) from July 2016 through June 2025^1^. Patients were excluded if baseline (pre-treatment) serum IL-6, hemoglobin, platelet count, albumin, serum creatinine, C-reactive protein (CRP), or immunoglobulin G (IgG) were missing, or if follow-up measurements of serum IL-6, hemoglobin, albumin, serum creatine, or CRP were missing. Severe iMCD were defined according to the CDCN severity classification^8^. Patients treated with siltuximab or tocilizumab—either as monotherapy or as part of combination therapy—were classified into the ‘anti-IL-6 therapies’ group, whereas patients who did not receive any IL-6-targeted drug were assigned to the ‘non-anti-IL-6 therapies’ group. Patients were classified according to the regimen active at the time of IL-6 measurement. This study was conducted in accordance with the Declaration of Helsinki and was approved by the Ethics Committee of PUMCH.

**Data collection**

Serum IL-6 concentrations were determined by a chemiluminescent immunoassay using the IL-6 assay kit (LK6P1; Siemens Healthcare Diagnostics Products Ltd.). All assays were performed in the central laboratory of Peking Union Medical College Hospital (PUMCH), and the same method was applied consistently across the entire cohort. At baseline (prior to the start of the current line of treatment), demographic information, histopathology, treatment details, symptoms, and relevant clinical, laboratory, and imaging data were collected. Follow-up data primarily included laboratory results and were assigned to the nearest scheduled time point (1, 3, 6, 9, 12, 15, 18, 21, or 24 months after treatment initiation). The above-mentioned data were collected until the occurrence of any one of the following events: discontinuation of the current line of treatment, switching to another therapy, loss to follow-up, or completion of 24-month follow-up after treatment.

**Assessment of treatment response**

Treatment response was assessed according to the CDCN biochemical criteria^8^. Complete response (CR) required normalization of CRP, hemoglobin, albumin, and estimated glomerular filtration rate (eGFR); partial response (PR) required > 50% improvement from baseline in each biomarker; stable disease (SD) was defined as < 50% improvement or < 25% worsening in all biomarkers; and progressive disease (PD) as > 25% worsening in any biomarker. Patients achieving PR or CR after the current treatment were classified as responders, whereas those with SD or PD were classified as non-responders. In this study, we focused on the current line of therapy and evaluated biochemical response specifically within that line. Treatment history was categorized as treatment-naïve (no prior iMCD-directed therapy), relapsed (prior PR/CR followed by PD), or refractory (patients who failed to achieve PR/CR with prior-line therapy). Event-free survival (EFS) was defined as the time from treatment initiation to the first occurrence of biochemical progressive disease, treatment-line switch, death, or last follow-up.

**Statistical analysis**

Analyses were performed with SPSS 30.0.0.0 and Python 3.13.5; figures were created in GraphPad Prism 10. Continuous variables are summarized as median with interquartile range (IQR) unless stated otherwise; categorical variables as n (%). Distributional normality was assessed with the Shapiro-Wilk test. All tests were two-sided with p < 0.05 considered statistically significant.

Baseline characteristics of patients receiving anti-IL-6 versus non-anti-IL-6 therapies were compared using χ² tests (or Fisher’s exact for sparse 2×2 tables) for categorical variables and t-tests when both groups were approximately normal or Mann-Whitney U otherwise for continuous variables. The Shapiro-Wilk test confirmed that serum IL-6 and the majority of continuous variables were not normally distributed. Associations between baseline serum IL-6 level and categorical covariates were tested with Mann-Whitney U (two levels) or Kruskal-Wallis (≥ 3 levels). Associations with continuous covariates were assessed using Spearman’s rank correlation (ρ).

For paired baseline-to-follow-up comparisons of logIL-6, we used a paired t-test if differences were normal or Wilcoxon signed-rank otherwise. Comparisons of IL-6 change at each time point (ΔlogIL-6 = logIL-6_time_ - logIL-6_baseline_) between different response groups were conducted with t-tests when both groups were normal and Mann-Whitney U otherwise. To test whether IL-6 trajectories differed by response after adjustment, we fit linear mixed-effects models with logIL-6 as the outcome; covariates included gender, age, clinical phenotype, histopathologic subtype, and severe iMCD status, with a random intercept for each patient. In patients receiving non-anti-IL-6 therapies, we assessed whether ΔlogIL-6 at each follow-up predicted response using receiver operating characteristic (ROC) analysis. The area under the ROC curve (AUC) and 95% confidence interval (95% CI) were estimated by the Hanley-McNeil method and the optimal cutoff was chosen by Youden’s J.

**a**

**b**

**Figure S1. Association between baseline IL-6 and treatment response**

Baseline logIL-6 did not differ significantly between responders and non-responders when all clinical phenotypes were analyzed together (a) or when restricted to the iMCD-IPL —the phenotype with the highest overall IL-6 levels (b) —regardless of treatment category (all therapies, anti-IL-6 only, or non-anti-IL-6 only).

iMCD, idiopathic multicentric Castleman disease; IPL, idiopathic plasmacytic lymphadenopathy; IL-6, interleukin-6; logIL-6, log10-transformed IL-6.

**a b**

**c d**

**Figure S2. Trajectories of serum IL-6 levels under anti-IL-6 and non-anti-IL-6 therapies excluding iMCD-TAFRO**

(a) Longitudinal change in IL-6 (ΔlogIL-6) for patients excluding iMCD-TAFRO. Anti-IL-6 therapy resulted in a modest increase in IL-6 levels above baseline (positive ΔlogIL-6) at most follow-up points (Table S5-1). In contrast, non-anti-IL-6 therapy led to a significant decrease from baseline across the follow-up points.

(b) Among responders only, anti-IL-6 therapies again caused an overall increase in IL-6 relative to baseline, whereas non-anti-IL-6 therapies exhibited a significant and sustained decrease.

(c) In patients receiving non-anti-IL-6 therapies, IL-6 levels decreased more substantially in responders than in non-responders, with statistically significant differences emerging as early as 1 month and persisting through 15 months (Table S5-3).

(d) Among patients who initially responded to non-anti-IL-6 therapies, those who maintained sustained remission continued to show suppressed IL-6 levels, whereas those who later experienced PD exhibited an early post-treatment IL-6 decline followed by a rebound.

PD, progressive disease; IL-6, interleukin-6; ΔlogIL-6, the change in log10-transformed IL-6 relative to baseline.

**Figure S3. Serum IL-6 trajectory in patients with disease progression after remission**

In patients receiving non-anti-IL-6 therapies who experienced PD after an initial response, IL-6 levels showed a rising trend before the occurrence of PD. Although non-significant, the upward trend in IL-6 levels began early, up to 3 months prior to the identification of PD.

PD, progressive disease; IL-6, interleukin-6; logIL-6, log10-transformed IL-6.

**Figure S4. Association between changes in IL-6 and laboratory tests in iMCD-IPL patients after non-anti-IL-6 treatment**

In iMCD-IPL patients receiving non-anti-IL-6 treatment, the decrease in IL-6 was significantly correlated with the recovery of hemoglobin and albumin, as well as the reduction in platelet count and IgG levels.

IL-6, interleukin-6; ΔlogIL-6, the change in log10-transformed IL-6 relative to baseline; IgG, immunoglobulin G.

**Figure S5. Two patients with transiently elevated CRP levels but stable IL-6 and other clinical markers**

Patient 1: A 47-year-old female with iMCD-NOS, treated with TCP, presented with fever on the day of the 9-month post-treatment follow-up. Computed tomography scans showed lung infiltrates suggestive of an infectious lesion. Although CRP transiently elevated, meeting the biochemical PD criteria according to CDCN standards, IL-6 and other laboratory markers such as hemoglobin, albumin, and creatinine remained stable. At the 12-month follow-up, the patient was still in biochemical CR.

Patient 2: A 34-year-old male with iMCD-NOS, treated with TCP, experienced significant transient elevations in CRP at the 6-month and 9-month follow-ups. Although the cause was not recorded, IL-6 and other laboratory markers, including hemoglobin, albumin, and creatinine, remained stable or continued to recover. At the 12-month follow-up, the patient remained in biochemical CR.
iMCD, idiopathic multicentric Castleman disease; NOS, not otherwise specified; TCP, thalidomide + cyclophosphamide + prednisone; PD, progressive disease; CR, complete response; CRP, C-reactive protein; logCRP, log10-transformed CRP; IL-6, interleukin-6; logIL-6, log10-transformed IL-6.

We additionally observed one patient with a transient concomitant rise in CRP and IL-6 during remission; however, IL-6 remained within the reference range and no other markers suggested relapse.

**Figure S6. Early IL-6 decline predicts subsequent biochemical response to non-anti-IL-6 therapies**

In patients receiving non-anti-IL-6 therapy, early post-treatment decreases in IL-6 (ΔlogIL-6) predicted later biochemical response. At 1 month after treatment, the ROC analysis showed AUC = 0.949 (95% CI, 0.888-1.000; p < 0.001); an optimal cutoff of ΔlogIL-6 = −0.16 yielded 91.7% sensitivity and 92.3% specificity. At 3 months, AUC = 0.889 (95% CI, 0.808-0.969; p < 0.001); a cutoff of −0.40 gave 72.5% sensitivity and 92.3% specificity.

IL-6, interleukin-6; ΔlogIL-6, the change in log10-transformed IL-6 relative to baseline; ROC, receiver operating characteristic analysis; AUC, area under the ROC curve; CI, confidence interval.


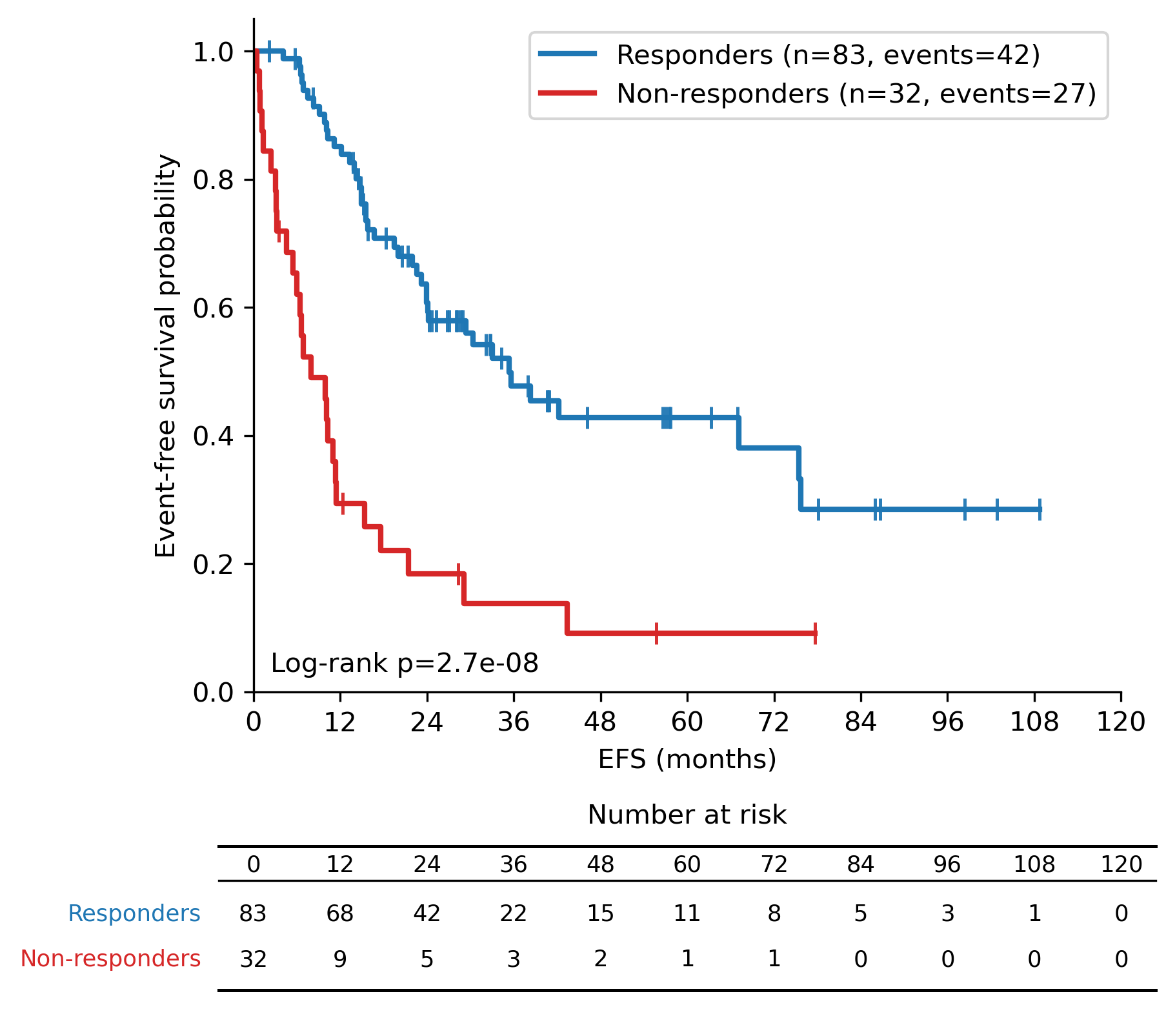


**Figure S7. Event-free survival in non-anti-IL-6-treated iMCD patients stratified by biochemical response**

To confirm the clinical relevance of biochemical response, we performed a Kaplan-Meier analysis of EFS stratified by response status in the non-anti-IL-6 group. Biochemical responders achieved a significantly longer EFS compared with non-responders (median 35.4 months [95% CI 23.9–75.5] vs. 8.0 months [95% CI 4.6–11.4]; log-rank p < 0.001; Figure S7). Event-free survival (EFS) was defined as the time from treatment initiation to the first occurrence of biochemical progressive disease, treatment-line switch, death, or last follow-up.

IL-6, interleukin-6.

**Table S1-1. Distribution of serum IL-6 measurements across follow-up timepoints in the iMCD cohort.**

| **Timepoint (months)** | **Anti-IL-6** | **Non-anti-IL-6** | **Patients still on same treatment** | **Switched treatment** | **Discontinued treatment** | **Lost to follow-up** |
| --- | --- | --- | --- | --- | --- | --- |
| **0 (baseline)** | 34 | 115 | 149 |  |  |  |
| **1** | 12 | 49 | 149 |  |  |  |
| **3** | 15 | 95 | 146 |  | 2 | 1 |
| **6** | 10 | 85 | 138 | 5 |  | 3 |
| **9** | 5 | 56 | 120 | 11 | 4 | 3 |
| **12** | 7 | 65 | 113 | 2 | 3 | 2 |
| **15** | 5 | 30 | 100 | 9 | 3 | 1 |
| **18** | 2 | 35 | 95 | 2 | 1 | 2 |
| **21** | 3 | 29 | 91 | 6 | 2 | 1 |
| **24** | 3 | 30 | 86 | 2 | 2 | 1 |
| **Total** | 96 | 589 |  | 37 | 17 | 14 |

149 iMCD patients were included, contributing 685 IL-6 measurements. Within each predefined time window, only one serum IL-6 measurement per patient was included; when multiple measurements were available, we selected the value closest to the nominal timepoint.

**Table S1-2. Clinical and histopathological subtypes and treatment distribution in the iMCD cohort.**

|  | **iMCD-NOS (*n* = 56)** | **iMCD-IPL (*n* = 74)** | **iMCD-TAFRO (*n* = 19)** |
| --- | --- | --- | --- |
| Histopathologic subtype |  |  |  |
| Plasmacytic | 42 (75.0%) | 67 (90.5%) | 4 (21.0%) |
| Hyaline vascular | 6 (10.7%) | 0 (0%) | 9 (47.4%) |
| Mixed | 8 (14.3%) | 7 (9.5%) | 6 (31.6%) |
| Treatment |  |  |  |
| Anti-IL-6 | 9 (16.1%) | 17 (23.0) | 8 (42.1) |
| Siltuximab-based | 9 | 17 | 8 |
| Tocilizumab-based | 0 | 0 | 0 |
| Non-anti-IL-6 | 47 (83.9%) | 57 (77.0%) | 11 (57.9%) |
| Thalidomide-based | 30 | 25 | 4 |
| Bortezomib-based | 14 | 28 | 7 |
| Sirolimus | 2 | 2 | 0 |
| Dara-D | 1 | 0 | 0 |
| BTK-inhibitor | 0 | 1 | 0 |
| R-CVP | 0 | 1 | 0 |

Data are presented as n (%). iMCD, idiopathic multicentric Castleman disease; NOS, not otherwise specified; IPL, idiopathic plasmacytic lymphadenopathy; TAFRO, thrombocytopenia, anasarca, fever, reticulin fibrosis / renal dysfunction, organomegaly; IL-6, interleukin-6; Dara-D, Daratumumab-dexamethasone; BTK, Bruton Tyrosine Kinase; R-CVP, rituximab, cyclophosphamide, vincristine, and prednisone.

**Table S2. Baseline characteristics of iMCD patients receiving anti-IL-6 and non-anti-IL-6 therapies.**

| **Characteristics** | **All (*n* = 149)** | **Anti-IL-6 (*n* = 34)** | **Non-anti-IL-6 (*n* = 115)** | ***p*** |
| --- | --- | --- | --- | --- |
| Gender-female | 73 (49.0%) | 20 (58.8%) | 53 (46.1%) | 0.192 |
| Age (range, year) | 45 (18-71) | 44 (24-63) | 45 (18-71) | 0.937 |
| Clinical phenotype |  |  |  | 0.065 |
| iMCD-NOS | 56 (37.6%) | 9 (26.5%) | 47 (40.9%) |  |
| iMCD-IPL | 74 (49.7%) | 17 (50.0%) | 57 (49.6%) |  |
| iMCD-TAFRO | 19 (12.8%) | 8 (23.5%) | 11 (9.6%) |  |
| Histopathologic subtype |  |  |  | 0.087 |
| Plasmacytic | 113 (75.8%) | 21 (61.8%) | 92 (80.0%) |  |
| Hyaline vascular | 15 (10.1%) | 5 (14.7%) | 10 (8.7%) |  |
| Mixed | 21 (14.1%) | 8 (23.5%) | 13 (11.3%) |  |
| Prior treatment |  |  |  | 0.155 |
| Treatment-naïve | 117 (78.5%) | 30 (88.2%) | 87 (75.7%) |  |
| Relapsed / Refractory | 32 (21.5%) | 4 (11.8%) | 28 (24.3%) |  |
| Severe iMCD | 44 (29.5%) | 11 (32.4%) | 33 (28.7%) | 0.681 |
| ECOG ≥ 2 | 31 (20.8%) | 5 (14.7%) | 26 (22.6%) | 0.319 |
| Constitutional symptoms | 126 (84.6%) | 31 (91.2%) | 95 (82.6%) | 0.288 |
| Organomegaly | 70 (47.0%) | 17 (50.0%) | 53 (46.1%) | 0.688 |
| Serous cavity effusion | 35 (23.5%) | 10 (29.4%) | 25 (21.7%) | 0.108 |
| Skin involvement | 25 (21.7%) | 15 (18.1%) | 10 (31.2%) | 0.354 |
| Lung involvement | 56 (37.6%) | 10 (29.4%) | 46 (40.0%) | 0.263 |
| IL-6 (pg/mL) | 24.4 (11.6-51.2) | 21.0 (12.4-41.2) | 27.6 (11.5-58.1) | 0.394 |
| Hemoglobin (g/L) | 95.0 (82.0-111.0) | 83.0 (77.2-97.8) | 97.0 (85.0-114.0) | 0.001 |
| Platelet count (10^9^/L) | 336.0 (208.0-444.0) | 334.0 (185.8-429.2) | 338.0 (218.5-448.5) | 0.327 |
| Albumin (g/L) | 32.0 (27.0-36.0) | 30.0 (25.2-34.0) | 32.0 (28.0-37.0) | 0.021 |
| Serum creatinine (μmol/L) | 65.0 (53.0-81.2) | 65.0 (49.2-104.5) | 65.0 (54.0-79.8) | 0.687 |
| CRP (mg/L) | 72.5 (31.9-129.7) | 71.4 (32.3-120.9) | 72.8 (32.7-132.1) | 0.696 |
| IgG (g/L) | 31.5 (17.6-45.8) | 33.0 (14.9-49.4) | 31.2 (18.7-44.4) | 0.918 |

Data are presented as median (interquartile range) or n (%). Severe iMCD and relapsed / refractory disease were defined according to the Castleman Disease Collaborative Network criteria^7^. Constitutional symptoms included fever, night sweats, fatigue, anorexia and weight loss. iMCD, idiopathic multicentric Castleman disease; TAFRO, thrombocytopenia, anasarca, fever, reticulin fibrosis / renal dysfunction, organomegaly; IPL, idiopathic plasmacytic lymphadenopathy; NOS, not otherwise specified; ECOG, Eastern Cooperative Oncology Group; IL-6, interleukin-6; CRP, C-reactive protein; IgG, immunoglobulin G.

**Table S3-1. Associations between baseline IL-6 and clinical characteristics**

| Characteristics | Category | *n* | LogIL-6 (pg/mL) | *p* |
| --- | --- | --- | --- | --- |
| Gender | Female | 73 | 1.4 (1.1-1.7) | 0.812 |
|  | Male | 76 | 1.4 (1.0-1.7) | |
| Clinical phenotype | iMCD-IPL | 74 | 1.6 (1.3-1.9) | < 0.001 |
|  | iMCD-NOS | 56 | 1.3 (1.0-1.6) | |
|  | iMCD-TAFRO | 19 | 0.9 (0.8-1.3) | |
| Histopathologic subtypes | Hyaline vascular | 15 | 0.9 (0.8-1.1) | 0.001 |
|  | Mixed | 21 | 1.3 (1.1-1.6) | |
|  | Plasmacytic | 113 | 1.5 (1.2-1.8) | |
| Prior treatment | Treatment-naïve | 117 | 1.4 (1.0-1.6) | 0.139 |
|  | Refractory / Relapsed | 16 | 1.5 (1.2-1.8) | |
| Severe iMCD | No | 105 | 1.4 (1.1-1.7) | 0.649 |
|  | Yes | 44 | 1.4 (0.9-1.7) | |
| Organomegaly | No | 79 | 1.4 (1.0-1.7) | 0.982 |
|  | Yes | 70 | 1.4 (1.1-1.7) | |
| Serous cavity effusion | No | 112 | 1.5 (1.2-1.8) | < 0.001 |
|  | Yes | 37 | 1.1 (0.9-1.4) | |
| Skin involvement | No | 114 | 1.4 (1.1-1.8) | 0.433 |
|  | Yes | 35 | 1.3 (1.1-1.6) | |
| Lung involvement | No | 93 | 1.4 (1.0-1.7) | 0.248 |
|  | Yes | 56 | 1.5 (1.2-1.8) | |
| Constitutional symptoms | No | 23 | 1.4 (1.0-1.7) | 0.711 |
|  | Yes | 126 | 1.4 (1.1-1.7) | |
| ECOG | 0-1 | 118 | 1.4 (1.1-1.6) | 0.436 |
|  | ≥2 | 31 | 1.5 (0.9-2.0) | |

Data are presented as median (interquartile range) or n (%). Severe iMCD and relapsed / refractory disease were defined according to the Castleman Disease Collaborative Network criteria^8^. Constitutional symptoms included fever, night sweats, fatigue, anorexia and weight loss. iMCD, idiopathic multicentric Castleman disease; TAFRO, thrombocytopenia, anasarca, fever, reticulin fibrosis / renal dysfunction, organomegaly; IPL, idiopathic plasmacytic lymphadenopathy; NOS, not otherwise specified; ECOG, Eastern Cooperative Oncology Group.

**Table S3-2. Correlations between baseline IL-6 and other laboratory parameters**

| Parameters | Spearman ρ | *p* |
| --- | --- | --- |
| Hemoglobin | -0.320 | < 0.001 |
| Platelet count | 0.476 | < 0.001 |
| Albumin | -0.453 | < 0.001 |
| Serum creatinine | -0.205 | 0.012 |
| CRP | 0.719 | < 0.001 |
| IgG | 0.294 | < 0.001 |

IL-6, interleukin-6; CRP, C-reactive protein; IgG, immunoglobulin G.

**Table S4-1. Trajectory of serum IL-6 in patients with anti-IL-6 and non-anti-IL-6 therapies**

|  | Anti-IL-6 |  | Non-anti-IL-6 |  |
| --- | --- | --- | --- | --- |
|  | logIL-6 (pg/ml) [95%CI] | *p* | logIL-6 (pg/ml) [95%CI] | *p* |
| 0 m | 1.38 [1.22, 1.55] (n=34) |  | 1.45 [1.36, 1.54] (n=115) |  |
| 1 m | 1.48 [1.18, 1.77] (n=12) | 0.182 | 1.14 [0.99, 1.29] (n=49) | < 0.001 |
| 3 m | 1.71 [1.40, 2.02] (n=15) | 0.006 | 0.97 [0.87, 1.07] (n=95) | < 0.001 |
| 6 m | 1.44 [1.00, 1.88] (n=10) | 0.178 | 0.90 [0.80, 1.00] (n=85) | < 0.001 |
| 9 m | 1.70 [0.87, 2.54] (n=5) | 0.341 | 0.77 [0.65, 0.90] (n=56) | < 0.001 |
| 12 m | 1.47 [0.94, 2.00] (n=7) | 0.555 | 0.74 [0.63, 0.84] (n=65) | < 0.001 |
| 15 m | 1.61 [1.12, 2.10] (n=5) | 0.312 | 0.88 [0.68, 1.09] (n=30) | < 0.001 |
| 18 m | 1.96 [0.77, 3.14] (n=2) | 0.5 | 0.82 [0.68, 0.96] (n=35) | < 0.001 |
| 21 m | 1.89 [1.33, 2.45] (n=3) | 0.56 | 0.76 [0.57, 0.95] (n=29) | < 0.001 |
| 24 m | 1.87 [0.83, 2.91] (n=3) | 0.863 | 0.78 [0.61, 0.94] (n=30) | < 0.001 |

**Table S4-2. Trajectory of serum IL-6 in responders and non-responders to anti-IL-6 therapies**

|  | Responders | Non-responders |  |
| --- | --- | --- | --- |
|  | ΔlogIL-6 (pg/ml) [95%CI] | | *p* |
| 1 month | 0.11 [-0.20, 0.41] (n=10) | 0.82 [-0.49, 2.13] (n=2) | 0.182 |
| 3 months | 0.35 [0.08, 0.62] (n=12) | 0.46 [-1.12, 2.04] (n=3) | 0.792 |
| 6 months | 0.17 [-0.36, 0.70] (n=8) | 0.73 [-0.71, 2.16] (n=2) | 0.400 |
| 9 months | 0.31 [-0.80, 1.42] (n=4) | 0.22 (n=1) | 0.800 |
| 12 months | -0.27 [-0.92, 0.38] (n=6) | 0.54 (n=1) | 0.571 |
| 15 months | -0.36 [-1.55, 0.83] (n=4) | -0.25 (n=1) | 0.800 |

**Table S4-3. Trajectory of serum IL-6 in responders and non-responders to non-anti-IL-6 therapies**

|  | Responders | Non-responders |  |
| --- | --- | --- | --- |
|  | ΔlogIL-6 (pg/ml) [95%CI] | | *p* |
| 1 month | -0.46 [-0.57, -0.36] (n=36) | 0.08 [-0.04, 0.20] (n=13) | < 0.001 |
| 3 months | -0.64 [-0.72, -0.55] (n=69) | -0.12 [-0.24, -0.00] (n=26) | < 0.001 |
| 6 months | -0.70 [-0.81, -0.59] (n=63) | -0.14 [-0.35, 0.06] (n=22) | < 0.001 |
| 9 months | -0.78 [-0.91, -0.66] (n=46) | -0.12 [-0.33, 0.08] (n=10) | < 0.001 |
| 12 months | -0.82 [-0.95, -0.69] (n=55) | -0.18 [-0.46, 0.10] (n=10) | < 0.001 |
| 15 months | -0.88 [-1.06, -0.69] (n=25) | 0.16 [-0.22, 0.54] (n=5) | < 0.001 |
| 18 months | -0.79 [-1.00, -0.58] (n=30) | -0.39 [-0.92, 0.14] (n=5) | 0.112 |
| 21 months | -0.73 [-0.94, -0.51] (n=25) | -0.05 [-0.53, 0.43] (n=4) | 0.009 |
| 24 months | -0.78 [-0.98, -0.58] (n=26) | -0.20 [-1.04, 0.65] (n=4) | 0.108 |

Data are presented as mean [95% CI] (number of valid data points). Paired baseline vs. follow-up timepoint analysis of log-IL-6 was performed using a paired t-test for normally distributed data, and the Wilcoxon signed-rank test for non-normally distributed data (Table S4-1). Comparisons of ΔlogIL-6 between responders and non-responders at each follow-up timepoint were performed using a t-test for normally distributed data, and the Mann-Whitney U test for non-normally distributed data (Table S4-2 and S4-3).

**Table S5-1. Trajectory of serum IL-6 in patients with anti-IL-6 and non-anti-IL-6 therapies excluding iMCD-TAFRO**

|  | Anti-IL-6 |  | Non-anti-IL-6 |  |
| --- | --- | --- | --- | --- |
|  | logIL-6 (pg/ml) [95%CI] | *p* | logIL-6 (pg/ml) [95%CI] | *p* |
| 0 m | 1.49 [1.30, 1.68] (n=26) |  | 1.50 [1.40, 1.59] (n=104) |  |
| 1 m | 1.69 [1.34, 2.04] (n=7) | 0.339 | 1.20 [1.04, 1.37] (n=42) | < 0.001 |
| 3 m | 1.86 [1.54, 2.18] (n=12) | 0.002 | 0.99 [0.88, 1.09] (n=90) | < 0.001 |
| 6 m | 1.60 [1.06, 2.14] (n=7) | 0.375 | 0.95 [0.85, 1.06] (n=77) | < 0.001 |
| 9 m | 1.99 [1.56, 2.41] (n=4) | 0.042 | 0.83 [0.70, 0.97] (n=49) | < 0.001 |
| 12 m | 1.69 [1.04, 2.34] (n=5) | 0.888 | 0.77 [0.66, 0.88] (n=60) | < 0.001 |
| 15 m | 1.76 [1.33, 2.18] (n=4) | 0.438 | 0.90 [0.69, 1.11] (n=29) | < 0.001 |
| 18 m | 1.96 [0.77, 3.14] (n=2) | 0.5 | 0.84 [0.70, 0.99] (n=32) | < 0.001 |
| 21 m | 1.89 [1.33, 2.45] (n=3) | 0.56 | 0.81 [0.60, 1.02] (n=25) | < 0.001 |
| 24 m | 1.87 [0.83, 2.91] (n=3) | 0.863 | 0.81 [0.64, 0.98] (n=28) | < 0.001 |

**Table S5-2. Trajectory of serum IL-6 in responders and non-responders to anti-IL-6 therapies excluding iMCD-TAFRO**

|  | Responders | Non-responders |  |
| --- | --- | --- | --- |
|  | ΔlogIL-6 (pg/ml) [95%CI] | | *p* |
| 1 month | 0.17 [-0.34, 0.68] (n=6) | 0.22 (n=1) | 0.857 |
| 3 months | 0.48 [0.22, 0.74] (n=9) | 0.46 [-1.12, 2.04] (n=3) | 0.964 |
| 6 months | 0.25 [-0.49, 1.00] (n=6) | 0.61 (n=1) | 1 |
| 9 months | 0.64 [-0.07, 1.35] (n=3) | 0.22 (n=1) | 0.5 |
| 12 months | -0.20 [-1.45, 1.05] (n=4) | 0.54 (n=1) | 0.8 |
| 15 months | -0.36 [-2.64, 1.91] (n=3) | -0.25 (n=1) | 1 |

**Table S5-3. Trajectory of serum IL-6 in responders and non-responders to non-anti-IL-6 therapies excluding iMCD-TAFRO**

|  | Responders | Non-responders |  |
| --- | --- | --- | --- |
|  | ΔlogIL-6 (pg/ml) [95%CI] | | *p* |
| 1 month | -0.48 [-0.61, -0.36] (n=31) | 0.07 [-0.07, 0.21] (n=11) | < 0.001 |
| 3 months | -0.63 [-0.72, -0.55] (n=66) | -0.13 [-0.26, -0.01] (n=24) | < 0.001 |
| 6 months | -0.71 [-0.83, -0.59] (n=56) | -0.13 [-0.34, 0.09] (n=21) | < 0.001 |
| 9 months | -0.80 [-0.94, -0.66] (n=40) | -0.12 [-0.35, 0.11] (n=9) | < 0.001 |
| 12 months | -0.84 [-0.97, -0.70] (n=50) | -0.18 [-0.46, 0.10] (n=10) | < 0.001 |
| 15 months | -0.89 [-1.08, -0.69] (n=24) | 0.16 [-0.22, 0.54] (n=5) | < 0.001 |
| 18 months | -0.83 [-1.06, -0.61] (n=27) | -0.39 [-0.92, 0.14] (n=5) | 0.086 |
| 21 months | -0.78 [-1.02, -0.54] (n=21) | -0.05 [-0.53, 0.43] (n=4) | 0.006 |
| 24 months | -0.80 [-1.01, -0.58] (n=24) | -0.20 [-1.04, 0.65] (n=4) | 0.102 |

Data are presented as mean [95% CI] (number of valid data points). Paired baseline vs. follow-up timepoint analysis of log-IL-6 was performed using a paired t-test for normally distributed data, and the Wilcoxon signed-rank test for non-normally distributed data (Table S5-1). Comparisons of ΔlogIL-6 between responders and non-responders at each follow-up timepoint were performed using a t-test for normally distributed data, and the Mann-Whitney U test for non-normally distributed data (Table S5-2 and S5-3).

**Table S6. Matched multivariable logistic regression for exacerbation in non-anti-IL-6-treated patients**

| **Predictor** | **OR (95% CI)** | *p* |
| --- | --- | --- |
| ΔlogIL-6 | 1.93 (0.24–15.42) | 0.535 |
| ΔlogCRP | 0.87 (0.19–4.09) | 0.862 |
| Sex | 0.57 (0.14–2.33) | 0.433 |
| Hemoglobin | 1.08 (1.01–1.16) | 0.019 |
| Albumin | 0.60 (0.44–0.83) | 0.002 |

ΔlogIL-6, the change in log10-transformed IL-6 relative to baseline; ΔlogCRP, the change in log10-transformed CRP relative to baseline.

**Table S7-1. Baseline serum IL-6 levels and association to treatment response of patients receiving thalidomide-based and bortezomib-based regimens**

|  | **All** | **Responders** | **Non-responders** | *p* |
| --- | --- | --- | --- | --- |
| **Thalidomide-based** | 28.6 (12.6-66.0) (n=59) | 27.6 (11.5-63.2) (n=43) | 39.3 (16.8-65.5) (n=16) | 0.603 |
| **Bortezomib-based** | 27.3 (11.0-51.2) (n=49) | 24.4 (9.9-50.5) (n=35) | 33.0 (14.2-64.8) (n=14) | 0.558 |

Baseline serum IL-6 levels did not differ significantly between the thalidomide-based and bortezomib-based subgroups (median 28.6 [IQR 12.6–66.0] vs. 27.3 [11.0–51.2] pg/mL; Mann-Whitney U, p = 0.661). IL-6, interleukin-6

**Table S7-2. Trajectory of serum IL-6 in responders and non-responders to thalidomide-based regimens**

|  | Responders | Non-responders |  |
| --- | --- | --- | --- |
|  | ΔlogIL-6 (pg/ml) [95%CI] | | *p* |
| 1 month | -0.47 [-0.64, -0.31] (n=20) | 0.09 [-0.13, 0.31] (n=6) | < 0.001 |
| 3 months | -0.63 [-0.75, -0.51] (n=35) | -0.01 [-0.11, 0.08] (n=14) | < 0.001 |
| 6 months | -0.66 [-0.81, -0.50] (n=31) | -0.02 [-0.19, 0.16] (n=12) | < 0.001 |
| 9 months | -0.75 [-0.91, -0.59] (n=26) | -0.03 [-0.32, 0.26] (n=5) | < 0.001 |
| 12 months | -0.89 [-1.07, -0.71] (n=28) | -0.16 [-0.60, 0.28] (n=7) | 0.005 |

**Table S7-3. Trajectory of serum IL-6 in responders and non-responders to bortezomib-based regimens**

|  | Responders | Non-responders |  |
| --- | --- | --- | --- |
|  | ΔlogIL-6 (pg/ml) [95%CI] | | *p* |
| 1 month | -0.47 [-0.63, -0.31] (n=15) | 0.03 [-0.18, 0.25] (n=6) | < 0.001 |
| 3 months | -0.67 [-0.80, -0.54] (n=29) | -0.28 [-0.51, -0.05] (n=11) | 0.004 |
| 6 months | -0.78 [-0.94, -0.62] (n=29) | -0.36 [-0.82, 0.10] (n=9) | 0.079 |
| 9 months | -0.81 [-1.03, -0.58] (n=18) | -0.22 [-0.61, 0.17] (n=5) | 0.012 |
| 12 months | -0.78 [-0.98, -0.58] (n=25) | -0.23 [-0.50, 0.04] (n=3) | < 0.001 |
